# Lectures on Clinical Surgery

**Published:** 1855-07

**Authors:** James Syme

**Affiliations:** Professor of Clinical Surgery in the University of Edinburg


Lectures on Clinical Surgery. By James Syme, Esq., Professor of Clinical Surgery in the University of Edinburg.

Lecture XI. *Tenotomy.*—The next case I shall bring before you is that of a boy with pes equinus or pointed toe. Before he is brought in, I may remark that, amongst the other advantages which Edinburg affords to medical students is, that we are not infested with specialists; we have here no cancer hospitals, no fistula infirmaries, no royal orthopœdic institutions, and you have consequently the opportunity of studying every variety of disease, while the patients are treated by practioners conversant with the whole range of surgery, and not by individuals whose only claim to confidence is their admitted ignorance of the general subject. Knowing the advantages of our position, both to the patient and the student, I feel it my duty to combat publicly the proceedings of specialists elsewhere. I know that in doing this I shall incur the charge of personality, as it is called; this, however, I do not regard; I neither know nor care for the individuals, it is their principles and their practice which I oppose. One of the most baneful departments of specialism is what is called "orthopœdy." The history of this subject is instructive. Of the various deformities to which the human frame is liable, those depending on contractions of tendons, or rather, of the muscles from which they arise, are amongst the most distressing. Until comparatively recent times, the only means of combatting these distortions was mechanical restraint, applied so as to stretch the parts into their natural form. These means were expensive and tedious in their application, and, still worse, very ineffectual; the ordinary course of proceeding being a painful struggle between the medical man and the deformity, till the patient, or rather his friends, (for he was generally very young,) gave up the contest in despair. You all recollect, I am sure, a remarkable instance of this in the person of a noble poet, whose career was probably greatly influenced by the deformity under which he labored, notwithstanding the most careful treatment employed in his childhood. Had that case occurred at the present day, there would have been no difficulty in its management. So, in the same way, many adults are to be seen with club-foot, who would have been free from deformity, had the improved practice now employed been in use when they were children. This great improvement depended on the introduction of subcutaneous incision for the division of tendons. In some cases, the free incision of contracted muscles had been used long before; for instance, in the case of wry-neck. This, however, was almost the only instance of its employment, and even here the results were not very satisfactory, the contraction of the cicatrix being apt to do away with the relaxation produced by the operation. The subcutaneous division of tendons, introduced within the last twenty-five years, was an improvement which every surgeon ought to take advantage of; but out of it has arisen a system of specialism in the deformities to which it is applicable. About twenty years ago, amongst the students in Berlin, was a young gentleman who had gone there from London, suffering from a form of club-foot which had existed congenitally. He was attending the lectures of Dieffenbach, and consulted that distinguished surgeon about his own case. Just at that time Stromeyer had introduced subcutaneous tenotomy, which was becoming a subject of attention; and Dieffenbach having advised him to go to Hanover in quest of relief, he accordingly went. The form of club-foot which he had was the pointed toe, with slight inversion of the foot. He had not been long away when, as Dieffenbach quaintly says, "In rushed the proprietor of the pes equinus, walking on the sole of his foot." Dieffenbach's attention being thus directed to the subject, he prosecuted it with his usual energy, and having a large field of observation, soon proved the high value of tenotomy. The gentleman who had been himself cured by it, was one of those employed by Dieffenbach in the inquiry; and, on his return to London, exerted all his influence to promote the adoption there of what he so well proved the advantage of, and succeeded in establishing an institution for the purpose of remedying deformities by tenotomy. This gentleman was Dr. Little, to whose proceedings, so far, I have no objection; but you will find that his institution, so far from discountenancing or abolishing the treatment which subcutaneous incision was proposed to supersede, has greatly promoted, and, indeed enormously extended the evils of that objectionable system. Various establishments, of a similar kind, have contributed to the same effect; and you have only to look into the books which their respective officers have published to see how completely the employment of mechanism has thrown into the shade the practice of tenotomy. I shall, probably, before long, have an opportunity of speaking more at length, and more emphatically, on this subject, with regard to lateral curvature of the spine. I will then show you how far the treatment of that disease has been thrown back by these orthopœdists, and how the practice of mechanical support, after being nearly abandoned, has, through them, been restored in all its integrity, and with tenfold force.

The boy, you will immediatley see, has pointed toe, and also some weakness of the limb. As regards the latter, the case is an example of the blight or partial palsy to which children are liable generally during teething. The child is observed to be hot and feverish for a few days, and on coming out of this state, is found to have lost power in one or more limbs; sometimes there is complete paralysis of half the body, longitudinally or transversely; in other cases, the affection is more limited. These attacks are curious, and not satisfactorily explained; but we must use every means of prevention, by removing causes of irritation, such as obstacles to the eruption of teeth, or intestinal disorder; for if once this condition has occurred, it is incurable; if it exists in any considerable degree, the patient remains for life a hopeless cripple; if it be only slight, it may perhaps be concealed, but never fully recovered from. In such cases the patient's friends are naturally anxious for relief; and when you honestly tell them that apparatus can do no good, they very likely take the child to quacks, and, through their confident assurances, are induced to spend much time and money in vain. It is, perhaps, natural that people should desire to employ mesmerism, friction, and other useless means; but if you have to do with reasonable persons, whose confidence you possess, you will be able to spare them all this anxiety and expense by telling them how the matter really stands.

[The boy, who was six years of age, and had had pes equinus for several pears, having been brought into the theater, Mr. Syme went on to say:—]

You observe when this boy walks the heel of his right foot does not touch the ground; also that the limb is somewhat smaller than the other. The mere smallness of the muscles is not, however, of itself necessarily evidence of the blight of which I was speaking; the want of use of the muscles which accompanies pointed toe accounts in part for it, and when the foot resumes its natural condition, the muscles will recover, to a certain extent, their proportions. In such a complicated case as this you might perhaps think it wrong to interfere; it appears, however, that it is proper to operate in cases like the present. You will not by so doing make matters worse in regard to strength; but will enable the powers that remain to be of more service to the patient. But if there is no power in the limb, it is of course useless to operate.

[Mr. Syme then divided the tendo-Achillis of the affected limb, and, having applied a piece of dry lint to the part and a figure-of-S bandage round the foot and ankle, said:—]

The bandage will be kept on for forty-eight hours, when no further treatment will be required, as the use of the limb in walking will be sufficient to bring the foot into its natural form. As to the mode of dividing the tendon, I would recommend you strongly to avoid cutting from within outwards, as recommended in books; nothing is more unreasonable in theory, or more inconvenient in practice. The easiest way is to put the tendon moderately on the stretch; then introduce between the skin and the tendon a narrow knife, with a pretty broad back, and a very keen edge, with the flat surface parallel to the skin; then turn the edge upon the tendon, and divide it by pressure over the back of the instrument, hardly using a sawing motion, else you will be apt to cut the skin. Double-edged knives, which have been recommended by some, are very inconvenient, as you cannot press upon them. The small wound made in the way I have recommended is sure to heal by first intention. There is, however, no operation in which it is so important to have the knife perfectly sharp, and the tough tendon soon takes off the edge; when the tendon is divided, a sudden snap informs you that your object is accomplished. This form of club-foot (so called, though not very properly) is very rarely congenital; of all deformities of the foot it is the most easily cured, and may generally be remedied even in adults without difficulty; whereas in cases of lateral displacement, unless you operate very early, a great deal of subsequant care is required in order to make the cure complete.

[On the third day after the operation the boy walked through the ward, putting his heel to the ground; and a day or two after, he was running about as usual, except that there was no tendency whatever to unnatural elevation of the heel. Mr. Syme showed him to the class, and remarked that the boy walked better than he could have expected, considering the partial blight of the limb. On the 26th of February Mr. Syme said:—]

I have now to show you a case of talipes varus of rather an unusual description. This brings us again in contact with the class of specialists who call themselves orthopœdists, and I once more beg to explain the objections I entertain to these practitioners. In the first place, they have endeavored to withdraw the treatment of deformities from the general practice of surgery, though constituting an important class of cases which are properly comprehended in it. Secondly, they have no claim whatever to the appropriation of these cases, the groundwork of orthopœdy being tenotomy, which is due, not to them, but to surgery in general. Five or six years before Dr. Little experienced in his own person the benefits of tenotomy at the hands of Stromeyer, I performed tenotomy in this place for wry-neck. Not that I was original in this respect, although I at the time supposed myself to be so; for though the operation had not before been performed in Great Britain or Ireland, it had been done ten years previously by Dr.

Dupuytren. Stromeyer was not a specialist, any more than Dupuytren or Dieffenbach, and therefore these specialists have no claim whatever to the discovery of tenotomy, which is the professed basis of their system. Thirdly, not being surgeons in a general sense, but having their attention confined to the specia subject, they do not perform the operations for the division of tendons properly, as we shall find from the work written by the head of their body. Fourthly, not being practical surgeons, and not having any great relish for operating, they are disposed to place the operation of tenotomy in the background, and substitute mechanical restraint for it. As I have already remarked, we are not infested here with these specialists, but I would advise you manfully and fearlessly to resist their encroachments wherever you may come in contact with them.

In this case you will immediately see, the inversion of the foot is to be removed, not in the old way by mechanical force, but by division of the tendons which are contracted. In cases of inversion of the foot, the tendon most frequently concerned is the tendo-Achillis, which, while the foot is in its natural position, and things are fairly balanced, draws the heel simply upwards, but if it be displaced outwards or upwards, will aggravate the deformity more than any other, on account of the power of the muscles inserted into it. Mr. Bishop endeavors to show that the tendo-Achillis being inserted into the outer rather than the inner part of the os calcis, its contraction must always tend to draw the foot outwards, and that therefore there can be no greater error than to divide the tendo-Achillis in cases of inversion.* Mr. Bishop's argument is not more sound than if he were to say that the muscles of a joint, such as the hip, because they tend to keep the bones in their place in the healthy condition, can therefore have no power in maintaining the displacement which has occurred in dislocation. So we find, in direct opposition to Mr. Bishop, that in almost every case of varus the tendo-Achillis requires to be divided. The tendon of the tibialis anticus is also nearly always contracted in talipes varus. Of the three tendons which run behind the internal malleolus, one (the tibialis posticus) has frequently a good deal to do with the production of inversion. The other two, the common flexor of the toes and the flexor longus pollicis, have little effect in producing inversion. The subject of these remarks is now before you. He is twelve years of age, and states that four years ago he fell upon the ice and sprained his ankle. He was treated at one of the dispensaries, where a liniment appears to have been rubbed along the course of the muscles of the leg; and after confinement to bed for about four weeks, he walked upon the outer side of the foot, as you see he still does.

---

* On Deformities of the Human Body. 1852.

You observe he rests his weight chiefly upon the cuboid bone, which is covered with a cushion, formed partly by hypertrophy of the integument, and partly by the thickening of a subcutaneoas bursa which naturally exists in this situation. The tendo-Achillis is in this case quite free from contraction, and so is that of the tibialis anticus; but on endeavoring to place the foot in its proper position, I distinctly feel the tendon of the tibialis posticus tightly stretched.

[Mr Syme then divided the tendon of the tibialis posticus a little below and anterior to the tip of the internal malleolus, the giving way of the tendon being indicated by a distinct snap. Having placed a small piece of folded lint over the wound, secured by a few turns of bandage round the ankle, he said:—]

This tendon is so distinctly located, that it is always discovered with facility in the situation where I have now divided it. It can generally be felt, and if not, you can hardly go astray; take the malleolus internus as your guide, and you are sure that the tendon lies a little below and anterior to it. Its position is well shown in this plate, (one of Colquet's.) If it be divided here, the worst that can happen will be, cutting the tendon of the common flexor of the toes, which can do no harm. The posterior tibial artery lies midway between the malleolus internus and the prominence of the calcaneum, and is, therefore, quite out of harm's way. * * * * * * * * * *

The case of the child with pointed toe, which you saw a fortnignt ago, reminds me of a girl with the same deformity, that was taken by its mother, a decent woman from Aberdeen, to an orthopœdic institution in London. She was there informed that there was no room in the establishment, but that she might be attended in private. She was very grateful for this; but afterwards learned to her dismay, that it would be necessary for her to pay ten guineas for the privilege of being attended as an outpatient. Being in poor circumstances, she was very much perplexed, until she heard of the arrival of a countryman—viz., myself, who had just gone to London, and consulted me. I admitted the patient into University College Hospital, divided the tendo-Achillis, and in a few days dismissed her to return home with the object of her long journey completely attained, though not precisely in the way proposed.

[In his lecture on the first of March, Mr. Syme showed to the class a boy, three years and a half old, affected with talipes varus, depending on contraction of the tendo-Achillis and the anterior and posterior tibial tendons of one foot, remarking that this was the most frequent form of talipes varus. Having divided the three tendons, and applied pieces of lint and a figure-of-8 bandage, he called attention to the fact, that the tendency to inversion was entirely removed. He then dismissed the child, with orders that it should be brought back in a few days. The boy with talipes varus caused by contraction of tendon of the tibials posticus alone, walked with his foot perfectly flat on the ground four days after the division of the tendon; but it then appeared that the great toe was drawn up to some extent through contraction of the extensor proprius pollicis, the tendon of which was prominent and tight on the dorsum of the foot. On the 5th of March, Mr. Syme showed the boy to his class, and divided the tendon of the extensor proprius pollicis, remarking that he had occasionally seen that tendon alone contracted, causing in some cases considerable inconvenience, and even serioosly interfering with walking, from the degree to which the great toe was drawn up. After division of the tendon, the toe assumed its natural position, and Mr. Syme therefore contented himself with the application of an ordinary bandage and pad of lint, instead of applying a splint to keep the toe down as he had sometimes found necessary. In the same lecture Mr. Syme said—]

I now wish to call your attention to a case of true talipes valgus, which is a very rare form of club-foot. The patient, who is a boy ten years of age, has had the deformity from his infancy, and I may remark that this affection is always congenital. There are four kinds of deformity of the foot, of which talipes varus is the most common. Next in frequency is the pes equinus, or pes caninus, as it ought more properly to be denominated, as the patient does not, like the horse, walk upon the extreme phalanx of a single digit, but upon the toes and their articulations with the metatarsal bones, like a dog. The other two forms are very rare, viz., talipes calcaneus, in which the toes are drawn up and the patient walks on the heel; and talipes valgus, or eversion of the foot. In valgus, as in varus, the tendo-Achillis may or may not be contracted, but the peroneal tendons are always too tight, and in the present case are alone affected. You observe the boy walks with a deformity like that produced by badly-treated fracture of the fibula, or at first sight you might suppose this to be an ordinary case of splay foot; but on looking attentively at it you observe that the arch of the foot is of the natural form, and that the deformity is that of simple eversion. You see, on the inner side of the foot, over the prominence of the navicular bone, a thickened bursa, produced by the pressure of the part against the shoe; and on the outer side the peroneal tendons form a tight prominent band below the malleolus externus, and their division will, I doubt not, afford complete relief.

[Mr. Syme then divided the tendons of the peronei muscles, below the outer malleous, and the usual dressing having been applied, the boy was taken back by his friends to Glasgow whence he had been brought the same day.

On the 8th of March, the child that had been operated on for talipes varus, by division of the three tendons on the 1st of March, was brought into the theater, and walked on the sole of his foot instead of on the outer side, as he had done before the operation; and his mother stated that he had been walking in the same way for the last five days. Mr. Syme called the attention of the class to the absence of all trace of the deformity, although no apparatus whatever had been employed, except the figure-of-8 bandage, which still remained as it was first applied, and which was now removed. On the same day, a boy, about eight years old, was brought to the hospital, affected with an extreme degree of talipes caninus, the metatarsal bones being in a line with the tibia. Mr. Syme remarked, that where the affection existed in this extreme degree, the astragalus sometimes became irremediably displaced as the patient grew up; but that in the present case, from the age of the child, he anticipated no such difficuly. Mr. Syme divided the tendo-Achillis at his lecture, and having placed the foot at a right angle, applied an ordinary bandage, and there could be little doubt that the boy would in a few days walk with a straight foot, as the others had done without any mechanical restraint.]

---

Lecture XII. *Amputation at the Ankle.*—The next case is that of a young man twenty-four years old, lying in Ward No. 5, who was admitted on the 12th of January, in a most pitiable state of suffering, being emaciated to an extreme degree, with a very rapid pulse, and his nervous system much disordered, as indicated by his tremulous tongue and vacant look. His right foot and ankle were much swollen, and an opening existed over the cuboid bone, which was soft and carious, the whole tarsus, indeed, appearing to be a mass of disease; an abscess on the lower part of the leg discharged, when opened, a quantity of thick, curdy substance, implying a very unhealthy state of the constitution. He stated that nearly twelve months ago a piece of iron fell on his foot, at the manufactory in which he was employed at Glasgow; swelling came on a week after the accident, and increased from that time forward till a fortnight before his admission, when an abscess in the foot was opened, and discharged a quantity of matter; yet he had kept on working, with frequent intermissions on account of the pain in his foot, till within the last six weeks. I should have amputated the foot at once, had I not thought that his general condition was so extremely bad that death must inevitably occur before long. We therefore contented ourselves with keeping the limb at rest, affording a free drain to the matter, and giving support to the system; but at the end of a month he was certainly not worse, and was very anxious for the operation; and having great confidence in the safety of the amputation at the ankle, I was induced to accede to his request. I may remark, that this is almost the only case in which the patient's constitutional state ever made me hesitate before resorting to this operation. The reasons why there is so little danger connected with it are, in the first place, that a very small portion of the body is removed; even part of the integument of the foot is left, viz., the skin of the heel, which forms the posterior flap, and the anterior flap, which extends a short distance over the dorsum of the foot; in fact little more is taken away than in Chopart's operation; and the shock to the system, so far as it depends upon the extent of the part removed, must consequently be less than from amputation of the leg. Another consideration is, that there are no large bloodvessels divided in the operation—only the anterior tibial, where it is much diminished in size, and the plantar branches being concerned; there is therefore little blood lost at the operation, or risk of serious secondary hemorrhage. In the third place, the tibai and fibula are divided through the cancellated texture, and you thus avoid the danger connected with opening the medullary hollow, especially where there is a disposition to phlebitis, while, at the same time, the spongy texture not being liable to exfoliation, like the dense part of the shaft, that inconvenience is prevented. On these grounds I anticipated that this operation would prove a very safe one; and this expectation has been now confirmed by long and very ample experience. Accordingly, I performed the operation in the present case, on the 13th of last month (February); and though I cannot undertake to say as yet that the patient is safe, yet he is certainly somewhat improved in general condition, and the stump is looking well. And, therefore, even if the patient should die, so far from this case leading you to think less favorably of amputation at the ankle, the improvement which has hitherto taken place will prove to you how very little disturbance of the constitution is produced by it. Patients, no doubt, may die after this amputation; but I believe they will hardly do so from the effects of the operation, if it be properly performed. In the first place, the incisions must be correctly made. A transverse one should be carried across the sole of the foot, from the tip of the external malleolus, or a little posterior to it (rather nearer the posterior than the anterior margin of the bone), to the opposite point on the inner side, which will be rather below the tip of the internal malleolus, but can be readily determined by placing the thumb and finger at opposite sides of the heel. If the incision be carried further forward, considerable inconvenience is experienced from the greater length of the flap; and I believe a great deal of the difficulty that has been attributed to the operation has arisen from this source—the operator getting into the hollow of the os calcis, cuts and haggles in striving to clear the prominence of the bone, with the desperate energy of an unfortunate mariner embayed on a lee shore in a gale of wind.

Another incision is then to be carried across the instep, joining the ends of the former. The next point to be attended to is, that in separating the flap of skin from the os colcis, you must cut parallel to the bone. This is of the greatest importance, since, when the flap is detached from the bone, its only supply of nourishment must be the branches which run through it parallel to the surface; and if, instead of keeping parallel to the surface you cut on the flap—as a butcher does when he skins a sheep—you will, by scoring it in this way, necessarily cut across these branches. I have reason to believe—nay, I know—that the sloughing which has occurred in some cases, has been due to these defects in the performance of the operation; the flap having been cut too long, difficulty has been experienced in separating it from the calcaneum, and this has led to the scoring of the flap, which has been inevitably followed by death of a portion or the whole of it.

The dressing is the next point to which I wish to allude. It is of great moment, particularly in a case like this, where the tissues of the flap are very soft and weak, to avoid as much as possible the application of pressure—tight bandaging would of itself be sufficient to kill the flap. I accordingly use a very light dressing for the first two days; and, in the present case, the flap was covered only with a piece of wet lint.

This is all I wish to say at present regarding the operation, but, before leaving the subject, I will make a few remarks upon certain alleged improvements which have been proposed. Some of these consist in differences with regard to the incisions, which have been made either diagonlly, with the view of taking the flap chiefly from the inner side, or so as to split the flap into two lateral halves. Both these modes of operating are sometimes necessary from the circumstances of the case, and I have myself had occasion to resort to both; but I think that any one who has tried either of them as well as the ordinary method will give decided preference to the latter, wherever it is practicable; and I cannot help believing that the very gentlemen who have proposed the diagonal and median incisions would themselves have suggested the transverse had it not been before in use. Not that I wish to be understood to imply that the suggestion of these modifications arose from a desire on the part of those who made them to differ from other persons; far from it; but regarding the operation of amputation at the ankle as a good one, and honestly striving to improve it, they have brought themselves to believe that the modifications above alluded to were really improvements. But the operation of a similar feeling would, I am convinced, have led much more readily to the suggestion of the transverse operation, had either of the others been that which was previously employed.

The next proposal to which I will allude is that of leaving the astragalus. My much respected friend, Dr. John Traill, of Arbroath, lately sent, through me, a communication on this subject, with an accompanying cast, to the Medi-Chirugical Society of Edinburg. Dr. Traill says very truly, that cases in which the astragalus could be left must very rarely occur, since disease affecting the tarsus at all generally involves the astragalus. But I think it would be very undesirable to leave this bone, even when it is not affected at the time of the operation, on account of its predisposition to caries; one of the great recommendations of amputation at the ankle being, that it removes all the bones which are liable to the disease. In Chopart's operation the calcaneum and astragalus are the only bones left, and though they appear perfectly sound at the time of the operation, yet the disease frequently recurs, as you may learn from the fact that I once performed three secondary operations, within the period of twelve months, upon stumps in which Chopart's operation had been practiced. The bones I hold in my hand were removed in one of these operations; and you will observe that, as in all other cases of the same kind, anchylosis has taken place between the astragalus and os calcis, while the latter bone has been previously drawn up by the action of the gastrocnemius, so as to prevent the patient from resting on the proper part of the stump. The same preparation shows the portions of the tibia and fibula that are removed in amputation at the ankle. They include, you see, the melleolar projections and a thin connecting slice of bone, merely sufficient to ensure removal of the articulating cartilage. In preserving the astragalus, therefore, you leave a bone which is peculiarly prone to disease, but there is another objection to this proposal. When you take away a foot, you must have in view the providing some substitute for it. It is true, the patient may walk without one, but practically it is necessary that between the leather of the boot that will be worn and the end of the stump a cushion of some soft elastic substance be interposed, and if the stump were as long as the sound limb it would not admit of this. Now, it appears that, when you amputate at the ankle in the ordinary way, the stump that results is just long enough to give the instrument makers sufficient room for the material of the artificial foot; so that if the astragalus be left the stump will be too long. Also, if yon retain the astragalus, the short, easily-formed flap of the ordinary operation will be insufficient to cover it, so that there will be danger of sloughing of the flap, as I before explained. For these reasons, therefore, I consider the preservation of the astragalus as undesirable.

The third and last modification is one lately proposed by Prof. Peregoff, of Petersburgh, which, on account of the distinguished source from which it emanated, has been tried in several quarters. The proposal is, to saw off the os calcis, leaving it attached to the integument of the posterior flap, and then turn it forwards, so as to make it uuite with the cut surface of the tibia. The objections to this method are, first, that the os calcis is a bone predisposed to caries, and it is therefore undesirable to leave any part of it; secondly, the operation is not facilitated by the change of procedure; but, on the contrary, considering the use of chain-saws, etc., involved, it is greatly complicated. Thirdly, the union of the osseous surfaces is not likely to take place easily. The fourth and principal objection is, that the integuments of the heel, which should form a cushion for the patient to rest on, are necessarily carried forwards, and the end of the stump is covered by skin, which is not designed for sustaining the weight of the body. I have, therefore, when consulted respecting the merits of this proposal, expressed a decided opinion against its adoption, and this advice has always been followed; but in some other cases, where it has been tried, the operators appear to have had reason for regretting that they did so. I may mention that often as amputation at the ankle has been performed for disease, and well established as it now is for those cases, a question still remained as to how far it was applicable in military surgery as a primary operation. I therefore requested my friend, Staff-Surgeon Dr. Gordon, who was surgeon of the 95th regiment, which you may recollect suffered so severely at the battle of the Alma, to state in writing his experience on this point. You are probably aware that in this campaign, from whatever reasons, wounds of the foot formed a large proportion of the injuries sustained. Dr. Gordon writes as follows: "I have much pleasure in stating that several cases of amputation of the foot (as first proposed and practiced by you) were performed after the battle of the Alma, and the results were most favorable. I am of opinion that your operation has many advantages, and, when adopted, will be the means of making a much more useful limb, independent of the less risk of life. I may likewise observe that those cases which came under my observation healed more rapidly than when the operation was performed at the lower third of the leg, and at the same time a much more useful stump was the result." Thus it appears that the operation is as valuable for injury on the battle field as it is for disease at home.

---

*Callous Ulcer of the Leg.*—The next case I shall bring before you is one which you may suppose entirely unworthy of your attention, belonging to common-place surgery, and of such a vulgar description as not to deserve discussion. It is one of ulcer of the leg, which, however, is no less adverse to the comfort of the patient than many diseases which are commonly considered more interesting, on account of some striking operation required in their treatment. Ulcers are derangements, with respect to which a great deal of misapprehension and bad practice have existed, and even now exist. In order to understand their treatment, it is necessary to have correct notions regarding their nature. When we talk of healing a sore, we do not mean that we repair it as we can a piece of furniture, since it is healed by a process of the animal economy, which is called granulation and cicatrization, all that the surgeon can do being to remove any obstacle that may be in the way of this process. It is plain that in order to discover such obstacles, and to remove them, we must understand the nature of the process; but it is a curious fact, that, notwithstanding the frequency of the granulating action in surgical practice, yet very erroneous ideas regarding it have long been very prevalent. You hear non-professional and often professional persons talking of an ulcer *filling up.* Long ago, when house-surgeon in this hospital, I had under observation a patient who had had a tumor removed from the axilla, and the cavity was granulating. I looked into the cavity as it grew smaller, and supposed it to be filling up; but the patient having died from some other cause, I found, to my great surprise, that the granulations formed but a thin pellicle over the natural textures beneath. What then had the wound been doing all the time? Soon after, a case occurred in which there was a large breach on the outer side of the forearm, exposing the radius and ulna; the sore granulated, and on putting my finger into it, I found a thin pellicle between the surface and the bone. I then happened to fall upon some papers in the Memoirs of the French Academy of Surgery—that valuable series of papers which contrast so strongly with the little edifying contributions of the same school at the present day. I there found the whole subject of granulation discussed fully and fairly. The fallacy of the notion of the filling-up of the cavity of a sore by granulation was clearly exposed, where, as the authors said, the same deception occurs as is experienced by a person gliding along in a boat on a smoothly-flowing river, who fancies the banks, the trees, etc., are moving past him, whereas it is really he that moves, not they; so, in watching a healing sore, you fancy the granulations are filling up, while in reality the integuments are descending. The first thing that takes place in a wound is the effusion of lymph, which next becomes red and vascular; the surface then becomes covered with pointed projections, called granulations; after which no further production of new material takes place, but an absorption, or, as the French surgeons say, an emaciation of surrounding parts, which allows them to be more and more easily drawn together as the granulating surface contracts. The diminution of the sore occurs most readily where the skin is loose; thus, in the case of sloughing the scrotum, a hardly perceptible cicatrix remains, whereas in less yielding parts, such as the palm of the hand, you see a considerable cicatrix formed over the remainder of the sore after contraction has occurred to the full extent permitted by the density of the textures. Keeping this in view, you will see that swelling of the surrounding parts will be a serious obstacle to healing. The true light in which the healing process should be regarded is not as reproduction, but as contraction.

I will now show you a patient who will exemplify what I am endeavoring to impress upon you. He tells that about twelve years ago he suffered an injury, which appears to have been fracture of the fibula of the left leg near the ankle; but he says that after being confined to bed for some weeks on account of it, he was able to walk as well as ever, and there was no deformity of the limb, the skin of which was not broken. Shortly after, when engaged in his occupation as a quarry-man, a small piece of stone flew against the left leg, a little above the ankle, striking it just over the prominent ridge of the tibai, where it made a little wound not half an inch in length, which inflamed, and became increased to a small ulcer. It remained about the same size, he says, till a year and a half ago, when he went to a horse doctor in Leith, who gave him some caustic substance to apply to the edge of the sore, and this had the effect of increasing its dimensions at every application, and he therefore abandoned its use. He has since continued his work as a quarry-man till two months ago, applying alum, red precipitate, etc., occasionally to the sore, which in the mean time increased in size till it attained its present dimensions. The ulcer, which has been carefully measured, is nine inches and a quarter across, and three inches in greatest verticle diameter; its surface is, you observe, almost smooth, and its edges are white and elevated nearly a quarter of an inch, while the surrounding parts are of brawny hardness. The affected leg is, you see, considerably swollen, measuring eleven inches in circumference at the lower margin of the sore, which is situated two inches and a half above the internal melleolus, while the right leg is only eight inches and a quarter round in the same situation. This is a good example of what is called the callous or indolent ulcer. Supposing Nature to be endeavoring, as she no doubt is, to heal the sore by the vis medicatrix, this brawny swelling must effectually oppose her efforts. The obstacle to healing is clearly only a local one; the patient is but forty-five years old, and his general health excellent, so that neither old age nor constitutional vice is to be contended with. This local obstacle is found in the swelling, which it is therefore our business to remove. The method formerly considered most effectual for this purpose was pressure, applied by strips of plaster covered with a bandage. This plan is still employed in some places, and is no doubt efficacious; but there are objections to it, on the grounds that it is expensive and troublesome in application, while recovery is tedious, and the patient is very liable to relapse. Hence this method is not very popular in public institutions, such as dispensaries and hospitals, the governors or managers of which are never fond of having such cases admitted. I am, therefore, happy to be able to tell you of another method which is very speedy in its action, and involves little trouble or expense.

Like many other good things in surgery, it was not the result of forethought, but of accident. I had a lady under my care five-and-twenty years ago, with a sore on the leg, which I was treating in the ordinary way; an attack of erysipelas came on, which I feared would retard recovery, but, on the contrary, when it subsided, the sore healed more rapidly than before. Attributing the beneficial result to the erysipelas, I thought the best way to promote the healing of an indolent ulcer would be to induce inflammation, and the most ready means of doing this seemed to be the application of a blister, which I accordingly used, with satisfactory results. A little consideration, however, sufficed to show that the blister did not act in any peculiar way, in producing this beneficial effect upon an ulcer, but only exerted its well-known power of causing absorption, as in case of chronic effusion into the knee-joint, where we know that a blister will with almost absolute certainty cause absorption. We shall apply a blister in the present case, and having taken careful measurements of the two limbs we shall be able to judge of its effect. The sore is adherent to the bone, which is an unfavorable circumstance, but I do not doubt that we shall succed in healing it completely.

The callous state of the surrounding parts is only one amongst various circumstances which may oppose the healing of sores, the names of which, such as "inflamed," "varicose," etc., have been given according to the nature of the impediment to recovery; but it will be time enough to speak of the other varieties of ulcer, when the cases present themselves. I omitted to mention how it is that the callous ulcer is produced. It is the effect of continued irritation upon a healthy sore. Inflammation from some cause siezes the leg, and when it passes off some swelling remains: repeated attacks of this kind make the sore deeper and deeper—not from the granulating pellicle descending, but from elevation of the surrounding parts. The effect of a blister is to remove this chronic inflammatory swelling, and you will probably see, in the present case, that, in a day or two after its application, the sore will be on a level with the surrounding skin, after which, simple dressing with lint soaked in water or some astringent lotion will be alone required. I may remark that this method has been employed in all parts of the habitable globe by my old pupils, who assure me that it has never failed or disappointed them.—*London Lancet.*

